# Ectoparasites may serve as vectors for the white-nose syndrome fungus

**DOI:** 10.1186/s13071-016-1302-2

**Published:** 2016-01-13

**Authors:** Radek K. Lučan, Hana Bandouchova, Tomáš Bartonička, Jiri Pikula, Alexandra Zahradníková, Jan Zukal, Natália Martínková

**Affiliations:** Department of Zoology, Faculty of Science, Charles University in Prague, Viničná 7, CZ-12844 Prague, Czech Republic; Department of Ecology and Diseases of Game, Fish and Bees, University of Veterinary and Pharmaceutical Sciences, Brno, Czech Republic; Department of Botany and Zoology, Masaryk University, Brno, Czech Republic; Institute of Molecular Physiology and Genetics, Slovak Academy of Sciences, Bratislava, Slovakia; Institute of Vertebrate Biology, Academy of Sciences of the Czech Republic, Brno, Czech Republic; Institute of Biostatistics and Analyses, Masaryk University, Brno, Czech Republic

**Keywords:** *Pseudogymnoascus destructans*, *Spinturnix*, Emerging infectious disease, Fungal infection, Vectors, Transmission

## Abstract

**Background:**

Vertebrate ectoparasites frequently play a role in transmission of infectious agents. *Pseudogymnoascus destructans* is a psychrophilic fungus known to cause white-nose syndrome (WNS), an emerging infectious disease of bats. It is transmitted with direct contact between bats or with contaminated environment. The aim of this study was to examine wing mites from the family Spinturnicidae parasitizing hibernating bats for the presence of *P. destructans* propagules as another possible transmission route.

**Methods:**

Wing mites collected from 33 bats at four hibernation sites in the Czech Republic were inspected for the presence and load of pathogen's DNA using quantitative PCR. Simultaneously, wing damage of inspected bats caused by WNS was quantified using ultraviolet light (UV) transillumination and the relationship between fungal load on wing mites and intensity of infection was subjected to correlation analysis.

**Results:**

All samples of wing mites were positive for the presence of DNA of *P. destructans*, indicating a high probability of their role in the transmission of the pathogen's propagules between bats.

**Conclusions:**

Mechanical transport of adhesive *P. destructans* spores and mycelium fragments on the body of spinturnicid mites is highly feasible. The specialised lifestyle of mites, i.e., living on bat wing membranes, the sites most typically affected by fungal growth, enables pathogen transport. Moreover, *P. destructans* metabolic traits suggest an ability to grow and sporulate on a range of organic substrates, including insects, which supports the possibility of growth on bat ectoparasites, at least in periods when bats roost in cold environments and enter torpor. In addition to transport of fungal propagules, mites may facilitate entry of fungal hyphae into the epidermis through injuries caused by biting.

## Background

White-nose syndrome is a virulent emerging fungal disease devastating bat populations across North America through disruptive effects on their hibernation [1–4]. It is caused by a psychrophilic fungus, *Pseudogymnoascus destructans,* found in Europe, Asia and North America, that grows on skin and wing membranes of affected bats, inflicting long-lasting injuries. In North America, these are a major cause of abnormal behaviour, resulting in depletion of fat reserves and consequent mass mortality [3, 5–7]. While WNS has resulted in a severe population decline in North America, there has been no significant mortality in Europe, despite wide distribution and high prevalence of WNS in many European bat hibernacula [8–11]. The morphology and high adhesivity of WNS fungus spores suggests that it is not capable of aerial transmission and that its spread among bats may be mediated through direct physical contact with infected individuals or through other indirect (e.g., environmental) sources of the pathogen [10, 12].

Arthropods that parasitize vertebrates, such as fleas, lice, ticks and mites, often play an important role in transmission of a range of pathogens, including agents of many emerging diseases [13, 14]. Mites of the genus *Spinturnix* are blood-feeding ectoparasites that live exclusively on wing membranes of bats and, in contrast to many other bat ectoparasites, stay on the bats’ body year-round, including the hibernation period [15]. Given their size (~1 mm) and horizontal transmission mode, wing mites may serve as an ideal vector of microscopic (~10 μm) spores and mycelium fragments of the WNS fungus. The aim of our study, therefore, was to detect the presence of *P. destructans* propagules, the agent of WNS, in spinturnicid mites obtained from hibernating bats.

## Methods

### Material collection

Using heat-sterilised forceps, we collected wing mites of the genus *Spinturnix* from 33 greater mouse-eared bats (*Myotis myotis*) at the end of the hibernation season (March - April 2014) at four sites in the Czech Republic, i.e., Kateřinská Cave (Moravian Karst), the Šimon and Juda Mine (Jeseniky Mountains) and the St. Kateřina and Kristína Mines (Šumava Mountains). The mites were determined morphologically using light microscopy [16, 17] in the pilot study as *Spinturnix myoti*. The sampled bat species did not host other *Spinturnix* taxa according to molecular genotyping [18].

In order to diagnose WNS and to quantify the fungus on bats, we swabbed the dorsal side of the extended left wing and transilluminated the left wing membrane of each bat using a UV lamp at 368 nm wavelength [19]. The wing was photographed and the number of fluorescent spots was counted from the photographs. Nine bats were not photographed due to technical problems in the mine. The total number of bats sampled for wing mites/swabbed/UV-examined at each site was: Kateřinská Cave (4/4/4), Šimon and Juda Mine (19/19/10), St. Kateřina Mine (6/6/6), Kristína Mine (4/4/4).

UV-guided wing biopsy punches [19], stored in formalin, were embedded in paraffin, cut to 5 μm tissue sections and stained with periodic acid-Schiff. Fungal skin lesions diagnostic for WNS were identified.

Additional swabs collected to cultivate *P. destructans* were transferred onto Sabuoraud agar and incubated in dark at 10 °C. Selected isolates were deposited into Culture Collection of Fungi, Prague, Czech Republic.

### Ethical approval

Sampling was performed in compliance with Czech Law No. 114/1992 on Nature and Landscape Protection, and was based on permits 01662/MK/2012S/00775/MK/2012, 866/JS/2012 and 00356/KK/2008/AOPK issued by the Agency for Nature Conservation and Landscape Protection of the Czech Republic. The authors are authorised to handle free-living bats according to Certificate of Competency No. CZ01297 (§17, Act No. 246/1992 Coll.).

### DNA isolation and quantitative PCR

One to 17 wing mites were sampled from each bat. All mites found on a single bat were pooled into a collection tube containing tissue lysis buffer (180 μl, Qiagen DNeasy Blood & Tissue Kit, Qiagen, Halden, Germany) and transported to the laboratory in a cooler. Directly after transfer, proteinase K (20 μl) was added to the samples and incubated at 56 °C for 2 h. Lysis buffer (200 μl) was added and the samples further incubated for 10 min. The manufacturer’s protocol was followed, and total isolated DNA was eluted in 100 μl of the elution buffer. DNA from swabs from dorsal side of the left wing was isolated with Qiagen QIAamp DNA Mini Kit (Qiagen) according to the manufacturer’s recommendation.

Fungal load on wing mites and bats was quantified with quantitative PCR (qPCR; [20]), using TaqMan® Universal Master Mix II with UNG (Uracil N-glycosylase; Life Technologies, Foster City, CA, USA). In order to optimise the PCR reaction, bovine serum albumin at final concentration of 0.05 mg/μl, and 0.025 U of Platinum® Taq DNA Polymerase were supplemented. Forward and reverse primers were used at a final concentration of 0.3 μM. Species-specific and genus-specific fluorescently labelled custom probes were used for the quantification of the PCR product, with final concentrations of 0.115 and 0.16 μM, respectively. The reaction mix was prepared on ice with 2 μl of DNA and three replicates were mixed for each DNA sample. Dual probes used in the Shuey et al.’s [20] protocol enable distinguishing true-positive samples with *P. destructans* from false-positives where high loads of related fungi occur [7].

A real-time qPCR reaction was performed on the LightCycler 480 PCR System (Life Technologies), with initial inactivation at 50 °C for 2 min and a hot start at 96 °C for 10 min. Nine cycles with a denaturation step at 95 °C for 15 s, and annealing at 62 °C for 1 min were followed by 43 identical cycles with quantification detection. The qPCR was finalised with a dissociation at 95–60–95 °C for 15 s each and cooled to 40 °C for 10 min. DNA isolated from a culture of the CCF3937 *P. destructans* strain [8] was used as a positive control and concentration reference during each run.

### Data analysis

A DNA concentration calibration curve was calculated from a dilution series of the CCF3937 *P. destructans* strain. The exact concentration of DNA in each dilution was determined using a Qubit HS fluorometer (Invitrogen, Carlsbad, CA), using the manufacturer’s protocol. qPCR effectivity was 1.96 and the sample concentration was calculated using custom scripts in R [21]. Fungal load was estimated from equation log (*q*_*PdDNA*_) = 3.194–0.287 *Cp*, *R*^2^ = 0.9719, where *q* is DNA concentration and *Cp* is the cycle. Each result was adjusted according to the positive control in its run and overall elution of the DNA. Fungal load on wing mites was calculated by dividing the obtained fungal load from the whole sample with the number of mites collected in that sample.

*Pseudogymnoascus destructans* load on the mites was correlated with the fungal load on the left wing and with the number of UV fluorescent spots diagnostic for WNS. The number of UV fluorescent spots corresponds with the amount of damage caused by the fungal infection [19].

### Results and discussion

**Fig. 1:**
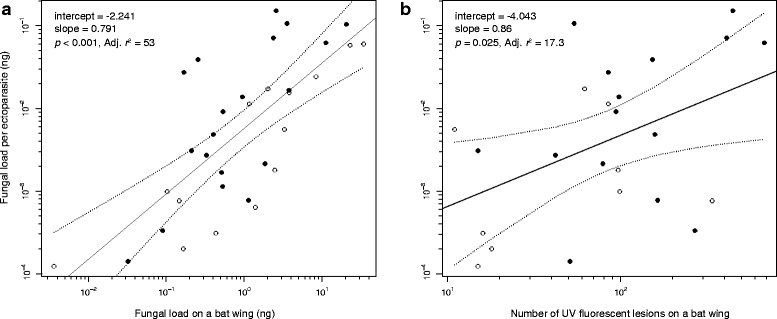
*Pseudogymnoascus destructans* load on ectoparasites. **a** Fungal load on wing mites in relation to *P. destructans* load on bat, and **b** number of UV-fluorescent spots representing WNS lesions, on the left wing. Both axes are log_10_. Solid line shows linear regression function, dashed lines delimit its 95 % confidence intervals

All 33 screened bats and all 33 wing mite samples were positive for *P. destructans* DNA. The fungal load sampled from bat wings was higher than that from ectoparasites (mean ± SD: *P. destructans* load on bat wing (ng) = 7.95 ± 15.26, *P. destructans* load on all mites sampled (pg) = 133.27 ± 170.37, *P. destructans* per mite (pg) = 49.38 ± 75.03). The fungal load on bat wing was significantly correlated both with fungal load on pooled wing mites (Pearson’s *r* = 0.69, *p* < 0.001) and with fungal load per wing mite (Pearson’s *r* = 0.74, *p* < 0.001; Fig. 1a). Furthermore, fungal load per wing mite was positively correlated with the number of fluorescent lesions on bat wings (Pearson’s *r* = 0.46, *p* = 0.02; Fig. 1b).

**Fig. 2:**
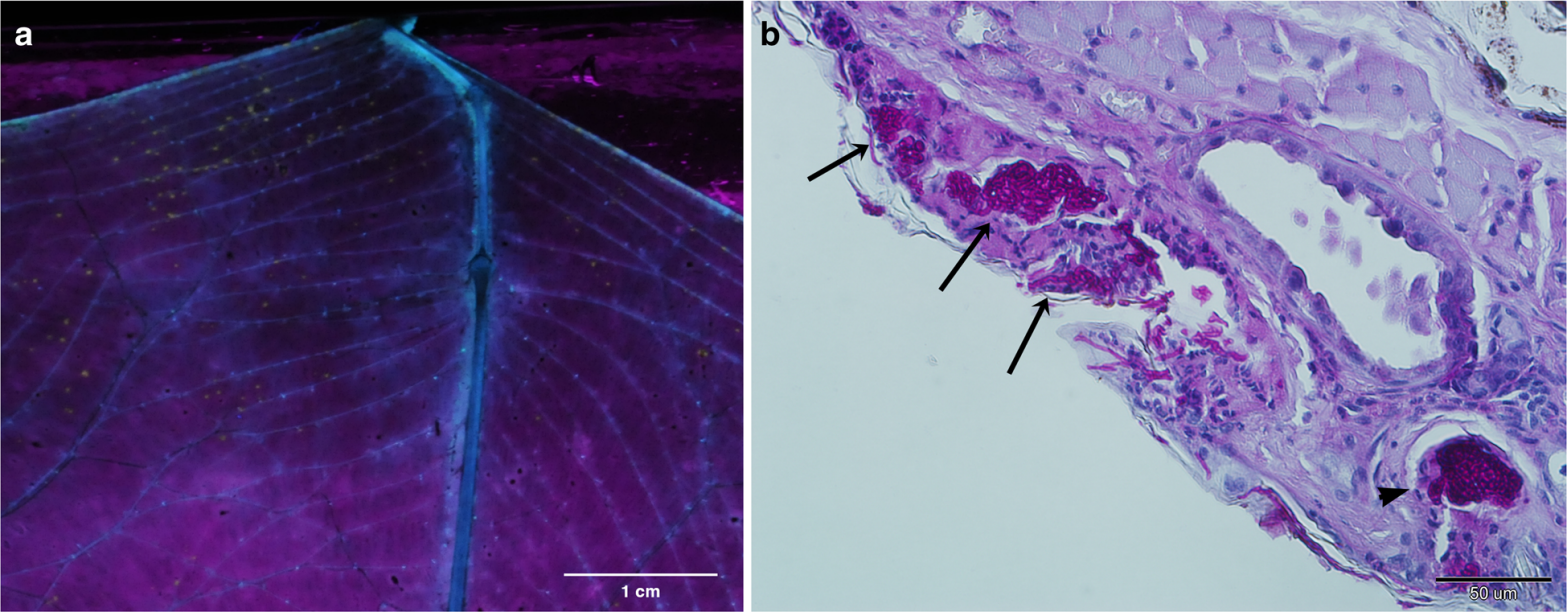
White-nose syndrome lesions on bat skin. **a** Orange-yellow spots displayed with ultraviolet light transillumination representing WNS skin lesions in *M. myotis* bat wing. **b** Histopathologic cross section from a UV-guided wing punch biopsy stained for fungi with periodic acid-Schiff shows three cupping erosions surrounded by necrotic tissue and neutrophilic infiltration (*arrows*) and fungal invasion in a hair follicle (*arrowhead*), confirming WNS

The sampled bats suffered from WNS as evidenced with the UV-fluorescent spots corresponding to WNS skin lesions (Fig. 2) that were found on all bats in this study. The pathogenic fungus was viable on them as proved by culture experiments (*P. destructans* isolates CCF4987-CCF4992).

Our data provide direct evidence for the presence of spores and/or hyphae from the fungus causing WNS on bat ectoparasites. Furthermore, we observed a positive relationship between amount of fungal infection on bats and fungal load present on wing mites.

Up until now, WNS fungus transmission has been assumed to occur between bats either by physical contact or by contact with the environment [22]. Given that spinturnicid mites switch hosts horizontally by crawling from one bat to another [23], our data demonstrate the potential for wing mites to play a role in the transmission of *P. destructans* spores between bats.

Ectoparasites in general play an important role as vectors of many diseases in vertebrates, including such emerging diseases as plague, malaria, leishmaniasis, trypanosomiasis, haemorrhagic fevers, babesiosis, borreliosis, tularemia, tick-borne encephalitis and many others [24, 25]. Unlike WNS, however, these are all caused by haemoparasitic agents, meaning that they are transmitted inside the bodies of the vectors. To our knowledge, there is no known disease transported by ectoparasite vectors on the outside of its body. Having said this, there are numerous examples of mechanical transport of pathogens by arthropods, such as the transport of rotaviruses, protozoan parasites or salmonellosis by non-biting flies and cockroaches [26–28]. We hypothesise that mechanical transport of *P. destructans* propagules between bats on the bodies of spinturnicid mites is enabled by their specialisation of living on bat wing membranes, i.e., the body region most typically affected by fungal growth [29]. This is supported by our finding of a positive relationship between fungal load on wing mites and fungal load and infection intensity on bat wings. An analogous situation may also hold true for other bat ectoparasites infesting bats during the time of fungal growth, such as Macronyssidae, fleas and nycteribiid flies; however, this remains to be investigated.

Positive findings of *P. destructans* in bat hibernacula over the summer period [30], and tests of its nutritional abilities, suggest that it is able to grow and sporulate on a variety of organic substrates, including dead fish, insects and mushroom tissues [31]. It is quite likely, therefore, that *P. destructans* can grow on bat ectoparasites, at least in periods when bats roost in cold environments and/or enter body torpor.

The transmission mode of wing mites, and their inability to survive off the host’s body, requires physical contact between bats [32]. Consequently, bat species that hibernate in clusters may be at higher risk of becoming infected by WNS than solitary hibernating bats. WNS prevalence is higher in bats forming clusters than in those hibernating solitarily, but solitary hibernators are also susceptible to the disease [11]. In this case, it is possible that *P. destructans* propagule transmission between bats takes place prior to hibernation, i.e., during the swarming period when bats are mating [33].

Last but not least, in addition to transport of fungal spores and/or fragments of mycelium, mites may facilitate entry of the fungal hyphae through the epidermis of bats via injuries caused by their bites. These injuries could prove very important for the pathogenesis of *P. destructans* skin infections as no signs of fungus keratinolytic activity were observed in the *stratum corneum* of bats under ultramicroscopy [34]. Our confirmation of the potential for wing mites to serve as vectors for *P. destructans* suggests a previously unknown transmission mode for WNS and stresses the importance of further research focused on testing this hypothesis.
